# An evaluation of a public partnership project between academic institutions and young people with Black African, Asian and Caribbean heritage

**DOI:** 10.1186/s40900-024-00564-4

**Published:** 2024-03-19

**Authors:** Alice Moult, Natalie Knight, Nathan Medina, Opeyemi Babatunde, Tom Kingstone, Helen Duffy, Kate Fryer, Krysia Canvin, Laura Swaithes, Lucy Brading, Lucy Bray, Wanda Russell, Krysia Dziedzic

**Affiliations:** 1 Impact Accelerator Unit, School of Medicine, Keele University, Newcastle-under-Lyme, ST5 5BG UK; 2 Eloquent Arts Centre, 41 Lichfield Road, Aston, B6 5RW UK; 3 School of Medicine, Keele University, Newcastle-under-Lyme, ST5 5BG UK; 4 Midlands Partnership University NHS Foundation Trust, Trust Headquarters, Corporation St, Stafford, UK; 5 Northern General Hospital, Herries Road, Sheffield, S5 7AU UK; 6 School of Medicine, Leeds Institute of Rheumatic and Musculoskeletal Medicine, University of Leeds, Worsley Building, Leeds, LS2 9JT UK; 7 Faculty of Health, Social Care and Medicine, Edge Hill University, Ormskirk, West Lancashire L39 4QP UK

**Keywords:** Patient and public involvement, Evaluation, Under-served communities, Young people, Black African and Caribbean heritage

## Abstract

**Background:**

This project (named Reinvent) aimed to promote Public Involvement (PI) in health research. Academics worked with a community group, the Eloquent Praise & Empowerment Dance Company, to develop a community partnership with young people from Black African, Asian and Caribbean heritage communities. The goal of this paper is to evaluate the Reinvent project for key learnings on how to engage and build partnerships with young people from Black African, Asian and Caribbean heritage communities.

**Methods:**

Reinvent developed a steering group which consisted of five young people, one academic, a Race Equality Ambassador and the Director of Eloquent. The steering group co-produced an agenda for two workshops and the evaluation tools used. The content of the workshops included drama exercises, discussions on physical and mental health, nutrition and school-life, short introductions to the concepts of research and PI, and group work to critique and improve a video currently used to promote PI in health research to young people. The evaluation tools included using the ‘Cube’ evaluation framework, video-blogging and collecting anonymous feedback.

Findings

The responses to the ‘Cube’ evaluation framework were positive across all four domains (agenda, voice, contribute change) in both workshops. A few of the young people described having a better understanding of the meaning and practice of PI in a video-blog. The anonymous feedback suggested that the workshops had increased young people’s confidence in sharing their thoughts and opinions about health and PI.

**Conclusion:**

Reinvent has shown that academic institutions and young people from an under-served community can partner to co-design workshops and apply evaluation tools. Working with young people in an environment in which they were comfortable, and by researchers joining in with the activities that the young people enjoyed (such as dance), enabled more informal and open conversations to develop. More work is needed to build upon this project so that young people can feel confident and supported to get involved in PI activities relating to research.

**Supplementary Information:**

The online version contains supplementary material available at 10.1186/s40900-024-00564-4.

## Abstract (highlights)

What were the aims of this project?

This project (named Reinvent) involved researchers working with the Eloquent Praise & Empowerment Dance Company to develop a partnership with young people from Black African, Asian and Caribbean heritage communities. The aim of this paper is to evaluate the Reinvent project for key learnings on how to engage with young people from Black African, Asian and Caribbean heritage communities in public involvement activities for research.

How did we do this project?

To plan the project we had a steering group with membership from young people from the dance company working alongside researchers. The steering group worked together to plan two workshops and agreed the tools to evaluate them. The content of the workshops included drama exercises, discussions on health, nutrition and school-life, short introductions to research and Public Involvement (PI) and group work to consider and improve a video promoting PI. The evaluation tools included answering four questions, video-blogging and collecting anonymous feedback using a post-box.

What did we learn?

The responses to the ‘Cube’ evaluation framework were positive across all four domains (agenda, voice, contribute change). A few of the young people described having a better understanding of the meaning and practice of PI in a video-blog. The anonymous feedback suggested that the workshops had increased young people’s confidence in sharing their thoughts and opinions about health and PI.

**Supplementary Information:**

The online version contains supplementary material available at 10.1186/s40900-024-00564-4.

## Background

Public Involvement (PI) is when research is carried out “with” or “by” patients and public contributors rather than “to”, “about” or “for” them [1]. PI is put into practice through people with lived experiences helping to inform and shape research in order to enhance study relevance, design, analysis, dissemination and governance [2–4]. Community-based participatory research (CBPR) is a research approach that emphasises working with communities to design and conduct research [5]. PI and CBPR share a philosophy of valuing partnership and collaboration [5]. The Kellogg Foundation Community Health Scholars Program define CBPR as involving all partners in a research topic of importance to the community with the aim of combining knowledge and action for social change [6]. CBPR also aims to include all relevant stakeholders as partners, rather than excluding for example health professionals from the process [7]. This contrasts with the PI approach in the United Kingdom (UK), which is informed by the National Institute for Health and Care Research (NIHR) and specifically excludes health professionals [8]. This has the benefit of making sure that public contributors are directly involved, rather than listening to professional voices as a proxy for lived experience. Taking this into account, this project takes a PI approach to developing and evaluating a partnership between academic institutions and young people from Black African, Asian and Caribbean heritage.

A challenge of PI is to ensure the inclusion of under-served communities, defined as people who use or might potentially use health or social services and who are less likely to be heard by these service professionals and decision-makers [9]. Whilst the optimal approach to achieving representation from these communities remains a topic of debate amongst the academic community [10], the UK’s NIHR suggests that the presence of diverse communities in health research fosters an inclusive environment, introduces a range of fresh skills and perspectives, and enhances research [11]. The NIHR has dedicated funding calls for developing innovative, inclusive and diverse public partnerships, in which this project was funded [12]. Furthermore, as part of their Equality Diversity and Inclusivity strategy [13], the NIHR developed a Race Equality Public Action Group (REPAG) for public involvement in research [14]. Hosted by the NIHR Centre for Engagement and Dissemination, the group aims to give people from Black African, Asian and Caribbean heritage communities - the terminology they chose - a stronger voice in shaping research priorities, the design and delivery of research, the recruitment of participants from these communities into studies, and the mobilisation of evidence into practice. REPAG developed a Race Equality Framework for Public Involvement in Research and we followed these principles of good practice throughout. REPAG suggested that people from these communities stress the need for organisations to develop trusting relationships with them to build their confidence in being able to contribute to research [14]. Research organisations can begin to build trust with under-served groups through community engagement. Community and charity groups can play a vital role in connecting researchers with under-served communities (145). These groups have already established trust-based relationships and often work creatively to support the communities with whom they work [16].

Layers of exclusion and intersecting inequalities can mean that young people (defined by the General Medical Council as those aged below eighteen years old [17]) from Black African, Asian and Caribbean heritage communities are even less likely to be involved in research and PI activities than their White counterparts [18]. There has been a growing call to include young people in decision-making regarding healthcare and health research to ensure that policies and services are relevant to their needs [19]. The involvement of young people in PI is important as it creates opportunities for them to influence the healthcare provided to them and expertise could contribute to addressing the needs of the community they represent [18, 20].

Public involvement activities in research can improve the appropriateness of a study for the wider target population to whom the research is relevant [21]. It is becoming increasingly important to evaluate PI to improve quality [22]. Critics of PI want more evidence of impact and those engaging with public contributors want to understand best practice [23]. For public contributors, through the process of evaluation, they can consider if there have been opportunities for meaningful involvement and researchers can gain insights into the contributors’ experiences [24].

### Partnership development

The Reinvent project, a term coined by the young people from the Eloquent, Praise & Empowerment Dance Company (henceforth referred to as Eloquent), was developed in partnership between Keele University, Sheffield University, Edge Hill University and Eloquent. The dance company provides a variety of activities ranging from dance classes, performing arts workshops, events, empowerment workshops, and mentoring. Over 150 young people, aged between four and eighteen years old, are currently enrolled in Eloquent.

Reinvent was also supported by a public contributor who is a member of the REPAG group and drama performer and teacher (NM), Keele University’s Race Equality Ambassador (NK) and the director of Eloquent. NK is also an established community representative at Eloquent and volunteers there on a weekly basis. Through these existing links, a funding application was developed and awarded (NIHR205207) to explore approaches to developing and evaluating a community partnership.

The Reinvent project aimed for academics to work alongside Eloquent to develop a community partnership with young people from Black African, Asian and Caribbean heritage communities. The specific aims of the project were:

- To co-design and run two workshops with the young people attending Eloquent
- To co-design an evaluation of each workshop with the young people attending Eloquent

It was anticipated that, depending on the success of the workshops, that the academic institutions and members of Eloquent would go on to co-produce a grant application on a topic that is important to the young people.

This paper presents the evaluation of the Reinvent project for key learnings on how to engage with young people from the Black African, Asian and Caribbean community for public involvement activities in research.

## Methods

This evaluation used a Concurrent Triangulation design [25] whereby both qualitative and quantitative content were collected and analysed concurrently and then compared to find areas of agreement and disagreement.

### Steering group

A steering group was convened and included five young people, one academic (AM), NK and the Director of Eloquent. The steering group believed that developing a partnership with academic institutions would provide learning opportunities about what ‘research’ is. This group met periodically throughout the project with NK also keeping in contact via instant messaging on a weekly basis. Instant messaging was the way in which staff at Eloquent communicated with the young people. At the beginning of the project the steering group met to agree the best way to establish a working relationship and decided that a co-productive approach could be taken. Co-production is a specific approach to PI whereby researchers and the public share power and decision making [26]. The notion of co-production was explained by NK and this approach seemed to fit the values that the young people thought were important. A key principle of co-production is reciprocity, which the steering group thought should be at the centre of this project. The young people were expected to gain new knowledge and would be remunerated for their involvement in the project. The researchers were expected to learn how to better engage with young people. The steering group thought that taking a co-productive approach was important to provide a platform to be heard. The steering group co-produced a structured agenda for each workshop (Tables 1 and 2). The group also helped to decide and co-produce the methods to evaluate each workshop.

**Table 1:** Workshop One planned agenda

Time	Title of session	Description of the session	Whole group or small group activities	Facilitator/s
11:00–11:10	Introductions	NK to introduce the academic members of the team to the young people, and provide a brief description of why the workshops were being conducted.	Whole group	NK
11:10–11:50	Drama exercises	As a ‘warm up’ session NM to introduce the young people to a number of drama exercises and games (e.g. Splat).	Whole group	NM
11:50-11:55	What is health and well-being?	NK to facilitate discussions on health and well-being.	Whole group	NK
11:50–12:25	Discussions on school life	NK, NM, AM, TK to facilitate discussions on school life.	The group splits into two groups based on age (12-15 years old and 16 years old and above). AM and NK to facilitate the younger group, TK and NM facilitated the older group.	NK NM AM NK
12:25-12:30	What is mental health?	NK to facilitate discussions on mental health.	Whole group	NK
12:30–13:30	Break (lunch provided)			
13:30–13:50	Drama exercises	As a ‘warm up’ session after lunch, NM to facilitate drama exercises and games (e.g. Splat).	Whole group	NM
13:50-13:55	What is research and how do we do it?	NK to facilitate discussions on research.	Whole group	NK
13:55–14:30	Discussions on nutrition and smoothie making	NK, NM, AM, TK to facilitate discussions on nutrition. The young people will also have to chance to make their own smoothies.	The group splits into two groups based on age (12-15 years old and 16 years old and above). AM and NK to facilitate the younger group, TK and NM facilitated the older group.	NK NM AM TK
14:30-14:35	What is Patient and Public Involvement and Engagement (PPIE?)	NK to facilitate discussions on PPIE.	Whole group	NK
14:35-14:55	Evaluation of the workshop	AM and NK to conduct the ‘Cube’ evaluation with the young people. • The young people will also have to chance to video-blog their experiences.	Whole group	AM, NK
14:55–15:00	Close of the workshop	NK to thank the young people	Whole group	NK

**Table 2:** Workshop two planned agenda

Time	Title of session	Description of the session	Whole group or small group activities	Facilitator
11:00–11:10	Introductions	NK to re-introduce the academic members of the team to the young people, and provide a brief description of why the workshops were being conducted.	Whole group	NK
11:10–11:50	Drama exercises	As a ‘warm up’ session NM to facilitate drama exercises and games (e.g. Splat).	Whole group	NM
11:50-12:00	What is Patient and Public Involvement and Engagement (PPIE)	NK to introduce the concepts of PPIE to the young people. NK to show a three minute video explaining what PPIE is.	Whole group	NK
12:00–12:30	Discussions on PPIE	NK, NM, AM, TK to facilitate discussions on PPIE.	The group splits into two groups based on age (12-15 years old and 16 years old and above). AM and NK to facilitate the younger group, TK and NM facilitated the older group.	NK NM AM TK
12:30–13:00	Break (light refreshments provided)			
13:00–14:00	Drama exercises	As a ‘warm up’ session after the break, NM to facilitate drama exercises and games (e.g. Splat).	Whole group	NM
14:00–14:30	Re-visiting discussions on nutrition and smoothie making	NK, NM, AM, TK to facilitate discussions on nutrition. The young people will also have to chance to make their own smoothies.	The group splits into two groups based on age (12-15 years old and 16 years old and above). AM and NK to facilitate the younger group, TK and NM facilitated the older group.	NK NM AM TK
14:30-15:00	Evaluation of the workshop	AM and NK to conduct the ‘Cube’ evaluation with the young people. The young people will also have to chance to video-blog their experiences.	Whole group	AM, NK
15:00–onwards	Close and a hot meal	NK to thank the young people. A hot meal was provided for all who attended.	Whole group	NK

The shorter version of the Gripp 2 (Additional file 1) was used to report our PI activities in relation to this evaluation, we provide more details on working with the steering group and also offer a critical reflection of the project.

### Ethical considerations

Every effort was made to provide a safe environment in which the young people were heard and supported. We followed safeguarding practices established within Eloquent which include the six main principles of safeguarding as outlined by the Care Act [27]; empowerment, prevention, protection, proportionality, partnerships and accountability. Additionally, within each workshop, the ethical approach was as followed:

1. Preparing carefully each session in advance, with specific information, tasks and/ or questions for the group in clear, accessible English; the language spoken by all of the young people.
2. Making it clear that the young person is under no obligation to take part in any element of the project, and could leave the session at any time.
3. Gaining consent from each young person and their guardian for each workshop.
4. Providing written information about the project, nature of the activity and contact details of NK.
5. Encouraging public contributors to discuss their involvement with their peers within and outside of Eloquent.

Young people were made aware that they could withdraw from any activity at any point and could discuss this with NK. The young people were remunerated via a voucher for each workshop. To support each other, researchers and NK met to debrief and reflect on the activities after each workshop.

It was agreed between the researchers and staff at Eloquent that the dance company would have ownership of the content created from the evaluation tools. NK verbally went through the findings of this manuscript with the young people, and staff from Eloquent were invited to review the article.

### Workshop design

The content of each workshop was co-produced with the steering group through regular discussions with NK. It was envisaged that each workshop would last approximately four hours and be held in-person at Eloquent’s headquarters in Birmingham. The steering group thought that drama exercises would be a creative way for the group to get to know each other and dissolve any hierarchies. The group also thought that physical and mental health, nutrition and school life were important topics to young people and should be discussed. Yet, the group also noted that the exact content should remain fluid and be changed in response to how engaged the young people were with the activities; this information was shared with the facilitators of each activity. Tables 1 and 2 describe the planned structure and content of Workshops One and Two respectively. Tables 3 and 4 describe the actual structure and content of the workshops. The facilitators and members of the steering group ultimately made changes on the day in light of ‘real-time’ feedback from the young people. NM developed the drama exercises and modified them in light of feedback from the steering group. Discussions on physical and mental health, nutrition and school-life were facilitated. NK gave a verbal introduction to the concept of health research and PI. A video created by staff at Keele University was shown and the young people were asked their opinions on how to improve the content.

**Table 3:** Workshop One actual agenda

Time	Title of session	Description of the session	Whole group or small group activities	Facilitator
11:15–11:20	Introductions	NK introduced the academic members of the team to the young people, and provided a brief description of why the workshops were being conducted.	Whole group	NK
11:20–12:20	Drama exercises	As a ‘warm up’ session NM introduced the young people to a number of drama exercises and games (e.g. Splat).	Whole group	NM
12:20-12:30	What is health and well-being?	NK facilitated discussions on health and well-being.	Whole group	NK
12:30–13:10	Discussions on school life	NK, NM, AM, TK facilitated discussions on school life.	The group splits into two groups based on age (12-15 years old and 16 years old and above). AM and NK to facilitate the younger group, TK and NM facilitated the older group.	NK NM AM TK
13:10–13:30	Break (light refreshments provided)			
13:30–13:40	What is mental health?	NK facilitated discussions on mental health.	Whole group	NK
13:40–14:10	Drama exercises	NM facilitated drama exercises and games (e.g. Splat).	Whole group	NK
14:10–14:40	Discussions on nutrition and smoothie making	NK, NM, AM, TK to facilitate discussions on nutrition. The young people will also have to chance to make their own smoothies.	The group splits into two groups based on age (12-15 years old and 16 years old and above). AM and NK to facilitate the younger group, TK and NM facilitated the older group.	NK NM AM TK
14: 40–15:00	Evaluation of the workshop	AM and NK conducted the ‘Cube’ evaluation with the young people. The young people had the chance to video-blog their experiences.	Whole group	AM, NK
15:00–onwards	Close and a hot meal	NK thanked the young people. A hot meal was provided for all who attended.	Whole group	NK

**Table 4:** Workshop Two actual agenda

Time	Title of session	Description of the session	Whole group or small group activities	Facilitator
11:00–11:10	Introductions	NK to reintroduced the academic members of the team to the young people, and provided a brief description of why the workshops were being conducted.	Whole group	NK
11:10–12:00	Drama exercises	As a ‘warm up’ session NM facilitated drama exercises and games (e.g. Splat).	Whole group	NM
12:00–12:10	What is Patient and Public Involvement and Engagement (PPIE)	NK introduced the concepts of PPIE to the young people. NK showed a three minute video explaining what PPIE is.	Whole group	NK
12:10–12:30	Discussing the PPIE video	NK, NM, AM, TK facilitated discussions on the PPIE video. The young people were invited to share their views on how the video could be improved.	The group splits into two groups based on age (12-15 years old and 16 years old and above). AM and NK to facilitate the younger group, TK and NM facilitated the older group.	NK NM AM TK
12:30–13:00	Break (light refreshments provided)			
13:00–14:00	Drama exercises	As a ‘warm up’ session after the break, NM facilitated drama exercises and games (e.g. Splat).	Whole group	NM
14:00–14:30	Re-visiting discussions on nutrition and smoothie making	NK, NM, AM, TK facilitated discussions on nutrition. The young people had chance to make their own smoothies.	The group splits into two groups based on age (12-15 years old and 16 years old and above). AM and NK to facilitate the younger group, TK and NM facilitated the older group.	NK NM AM TK
14:30-15:00	Evaluation of the workshop	AM and NK conducted the ‘Cube’ evaluation with the young people. The young people had the chance to video-blog their experiences.	Whole group	AM, NK
15:00–onwards	Close and a hot meal	NK thanked the young people. A hot meal was provided for all who attended.	Whole group	NK

### Involvement strategy

The young people were recruited to the workshops via NK. Within her voluntary capacity, NK verbally told the young people who regularly attended Eloquent about the workshops and a written invitation was sent out to all young people via Eloquent’s monthly newsletter. The steering group also discussed the project with their peers. To register their attendance for each workshop the young people had to verbally express an interest to NK and complete and return a consent form; the parent also had to sign this consent form. The consent form was the standard consent form used by Eloquent. The steering group thought that this process of consent was familiar to the young people and their guardians.

### Evaluation tools

The evaluation methods were co-produced by the steering group and included: completing the ‘Cube’ evaluation framework [28], video-blogging (vlogging) and posting anonymous feedback into a post-box within Eloquent’s headquarters during Workshop Two.

### The ‘Cube’ evaluation framework

The ‘Cube’ evaluation framework [28] is a theoretical model which takes into account the dynamic and fluid nature of social interactions [28]. The four dimensions, and each question asked in relation to each of the dimensions, are detailed in Table 5. The steering group helped to develop each question to ensure relevance to the project and young people.

**Table 5:** The four dimensions of the ‘Cube’ evaluation framework

Dimension	Description	Questions asked within the Workshops
Voice	Strong voices discuss issues and influence decision-making. Weak voices may discuss issues, but have little influence on decision-making.	Did you feel like your voice was heard?
Contribute	Knowledge can take on different forms, which may not be equally valued. A single involvement approach is likely to privilege one social/cultural group over another, thus perpetuating inequality.	Did you feel like there were enough activities to get involved in?
Agenda	Public concerns are in the context of social action, e.g. public opinion, norms and values, as well as individual experiences and behaviours. Organisation’s concerns are, e.g. bureaucracies and markets.	Did you feel like the event was based on things that mattered to you?
Change	Decision-makers’ willingness and ability to respond to issues raised by participants in knowledge spaces depend on contextual factors, e.g. economic resources and national policies.	Did you feel like the facilitators listened to you?

Within each workshop, NK introduced the framework and questions. The young people were then asked to map their experiences of being involved within the workshops along the four dimensions. Each dimension was separately represented on a wall chart. The steering group suggested offering the young people the response categories of ‘yes’, ‘no’ or ‘maybe’ along a continuum on the wall chart. These continuum measures corresponded to high/ medium/ low scores. The young people were asked to use a sticky note to indicate which category they felt best represented their experiences. The young people were also asked to write comments on their sticky notes explaining their choice. NK made it clear that the young people did not have to complete this activity if they did not wish to. Within Workshop One the planned allocated time for completing this activity was 20 minutes, however, the prior activities within the workshop overran and the evaluation had to be completed in 10 minutes. Within Workshop Two 30 minutes was dedicated to completing the ‘Cube’ activity.

### Video blogs

The steering group suggested having one young person as the ‘interviewer’ who asked four questions to other young people. The four questions were: (i) What did you learn from today’s workshop? (ii) Do you have any new insights from today’s workshop? (iii) What was your favourite part of the workshop? (iv) What would you like to see us do at the next workshop? The same questions were asked within both workshops.

Within Workshop One the vlogs lasted a total of 15 minutes. Within workshop Two the vlogs lasted 25 minutes. The vlogs were captured on a video-recorder and the MP4 files were transferred to a computer.

### Anonymous feedback

Following Workshop One, and in light of the feedback from the steering group, an anonymous post-box was placed within the room in which Workshop Two was taking place. The young people were invited to write feedback and reflections on both of the workshops and post them in the post-box.

## Analysis

The content from each evaluation tool were analysed separately and then compared to each other to provide a more comprehensive understanding of the partnership.

### The ‘Cube’ evaluation

After each session the wall charts displaying each question relating to each domain of the ‘Cube’ were photographed and collated. Researchers looked at each photograph and counted each sticky note in the ‘yes’, ‘no’ or ‘maybe’ categories relating to each question for each workshop. The researchers developed a table which documented the responses for each domain.

### Video blogging

The researchers translated the vlogs into written form and were first analysed by AM and NK who identified common themes. These themes were discussed and modified by the wider research team and sense-checked with the steering group.

### Anonymous feedback

Each piece of feedback was counted and translated into written form. AM and NK first identified common themes which were discussed and modified by the wider research team and sense-checked with the steering group.

## Findings

The first workshop was held on the 30th May 2023 and was attended by 25 young people; six males and 19 females. The ages ranged from between 13 and 17 years old. Two academics (AM, TK), a public co-applicant (NM), NK and the Director of the Eloquent attended.

The second workshop was held on the 26th July 2023 and was attended by 16 young people; three males and 13 females. The ages ranged from between 13 and 17 years old. All of the young people who attended the second workshop had been present at the first workshop. Two academics (AM, OB), a public co-applicant (NM), NK and the Director of the Eloquent attended.

### Summary of the ‘Cube’ results

A low proportion of young people who attended Workshop One completed the ‘Cube’ (five/six out of 25), whilst a high proportion of young people who attended Workshop Two completed the activity [15 out of 16]. The increase in the completion of the ‘Cube’ may have been because there was increased time dedicated to this activity.

Overall, results were positive across all four dimensions within both workshops. There were zero scores for ‘no’ across both workshops. The ‘agenda’ dimension within Workshop Two had the most scores relating to ‘maybe’. Table 6 details the responses relating to each dimension. No comments were written on the sticky notes within either workshop.

**Table 6:** Responses to the ‘Cube’ framework

Workshop One	The ‘Cube’ dimension	Yes (%)	Maybe (%)	No (%)	Total number of responses
	Voice	83.33 (n=5)	16.67 (n=1)	0	6
	Contribute	100 (n=6)	0	0	6
	Agenda	80 (n=4)	20 ( =1)	0	5
	Change	100 (n=6)	0	0	6
Workshop Two	Voice	100 (n=15)	0	0	15
	Contribute	86.67 (n=13)	13.333 (n=2)	0	15
	Agenda	80 (n=12)	20 (n=3)	0	15
	Change	100 (n=15)	0	0	15
*Numbers are presented as a percentage of the total number of responses					

### Summary of video-blogs

Within Workshop One, one young person was the interviewer and four young people answered questions. Within Workshop Two, one young person was the interviewer and six young people were interviewed.

All of the young people who attended both workshops voiced that they had developed self-confidence when participating in the whole and smaller group activities, and had been able to use their voice to speak their mind and to be their authentic self. The topics of discussion in both workshops were described as relevant to young people. Some of the young people within the video-blogs suggested that they have new insights into how to express emotions because of these discussions. A few of the young people described the meaning and practice of PI.

All of the young people described that their favourite activities within each workshop were the drama exercises as these permitted them to bond as a group and to gain a better understanding of everybody’s personalities. All of the young people described that they enjoyed the format of the workshops and would attend another; they proposed a topic for the group work could focus on how to manage a hobby (such as dance) along-side school-life.

To improve the workshops, the young people recommended including dance and singing exercises, as well as drama. All young people also thought that the workshops should be whole day events.

### Summary of anonymous feedback

There were a total of 25 notes which contained anonymous feedback. The anonymous feedback mainly centred on what the young people had learnt during the workshops. Learning was discussed in terms of them being a performer and gaining personal skills. As a performer, the activities facilitated by NM helped the young people to think about being louder, having a better physical stance and how to present themselves when in a group. On a personal level, the young people described that the workshops helped them to communicate, improved their teamwork and increased their confidence in using their voice to share their thoughts and opinions about health and PI.

Many of the comments also included references to feeling more understood about their struggles relating to school. A few comments indicated that the young people felt safe to discuss potentially sensitive topics; one comment described how a young person had been bullied at school and how the workshops made them feel like they did not have to change who they are. A few comments proposed that the workshops should have been whole day events as some of the activities seemed rushed.

### Discussion

This manuscript has presented an evaluation of the Reinvent project. The evaluation tools included using the ‘Cube’ evaluation framework, video-blogging and collecting anonymous feedback. The responses to the ‘Cube’ evaluation framework were positive across all four domains (agenda, voice, contribute change) in both workshops. A few of the young people described having a better understanding of the meaning and practice of PI in a video-blog. The anonymous feedback suggested that the workshops had increased young people’s confidence in sharing their thoughts and opinions about health and PI.

The ‘agenda’ dimension within Workshop Two had the most scores relating to ‘maybe’; this could have been due to the demographics of the steering group—all of whom were female. In retrospect, a more diverse steering group could have ensured that the content of the workshops were important to a variety of young people.

The partnership between the universities involved in this project and Eloquent was strategic in nature as NK has been a volunteer at Eloquent for over a decade. NK had a boundary spanning role both working in the Impact Accelerator Unit, Keele, and being a community representative for Eloquent. Due to time resources that would be needed to begin to develop a community partnership, partnering with Eloquent offered pragmatic advantages for engaging with young people from a community under-served by health research. Collaborating with a community representative external to the research team has been previously recommended [29], however, we found that NK’s boundary spanning role enabled her to have both knowledge of the academic environment and community partner, thus providing a strong communication link between the two. The role adopted by NK in this project may also have helped to foster ‘ethically conscious’ activities by helping to establish relationships and rapport, enabling the young people to experience safe boundaries and a space to disclose personal experiences.

### Community interests

Whilst the workshops were co-designed with a steering group, it is clear that the planned activities changed on the day of each workshop. This was in response to feedback from the young people. Previous research has suggested that academics should avoid overly rigid and inflexible activities [30]. Researchers with limited experiences of conducting such workshops may feel that they need to heavily structure activities to feel confident that all information gathering will be covered [30]. This project has shown that academics should approach such activities with an openness to respond to what is heard from the young people and build in a contingency plan if anything unexpected happens. By doing this researchers are ensuring that the community’s topics of interest are central.

### The impact upon the young people

Within the PI field there is a recognition of the need to understand the impact of involvement activities [31]. Impact is one of the six UK Standards for Public Involvement in research which aim to help researchers and organisations improve the quality of the public involvement [32]. The evaluation methods highlighted the impact and value of the workshops in increasing young peoples’ confidence in sharing their thoughts and opinions in a group setting. More work is needed to assess if the impact of these workshops results in young people having the confidence to pursue their own involvement in PI activities and research.

### Evaluation methods

Previous studies have undertaken different approaches to evaluating PI activities primarily at the end of their research activities [33]. The steering group wished for each workshop to be evaluated separately, but via multiple methods. By doing this and sharing learning from the first workshop (e.g. that the young people wanted more drama exercise activities), it maximised the impact on shaping the delivery of the second workshop. It is clear that multiple methods are emerging to support the evaluation of public involvement [23]; researchers are recognising that there is no ‘one size fits all’ method. By using multiple evaluation methodologies the project has gone some way to optimise learning both individually for the young people, and collectively as a research team. The use of video blogs to capture the experiences of the young people was innovative and novel, however, this method needed time to set-up and finances to purchase the relevant equipment. Vlogging was also time-consuming during the workshops meaning that not all young people were able to be involved. More research is needed to understand how vlogging could work in practice whilst offering inclusive opportunities for all involved within the activity.

Previous research has used the ‘Cube’ framework to evaluate PI activities [34]. The benefits of using this framework were that it enabled cross-sectional comparisons between the two workshops and the results were immediately available allowing for activities to be modified in a timely manner. The lack of responses in the first workshop was an important indicator that the young people needed more time to complete the exercise in the second workshop. Studies which have asked adults to write narrative feedback when completing the ‘Cube’ suggested that it encouraged public contributors to reflect about their involvement and experiences [28]. The young people in this project provided no narrative feedback when completing the ‘Cube’, but did provide a number of comments via an anonymous post-box showing the importance of using multiple evaluation methods. This project suggests that young people may value the anonymous nature of evaluating PI activities. The completion of the ‘Cube’ did permit the academics to reflect on the purpose and strategic directions of the workshops and could help to inform the planning of future PI activities.

### Recommendations

Table 7 describes the recommendations from this evaluation when working with young people from an under-served group.

**Table 7:** Recommendations

1) To develop a diverse steering group to ensure representation of young people with differing demographics
2) Creative methods of engagement are key to partnership building with young people, however, these methods need to be carefully co-produced with researchers and young people
3) To acknowledge the importance, and assess the impact, of the boundary spanning roles of some individuals (e.g. those who have links with both academia and community groups)
4) To be responsive to the young people during workshops to ensure their interests are central; this may mean being flexible in terms of activities
5) To research the impact of developing partnerships and if these partnerships do, or do not, result in more involvement and engagement within PI and research activities
6) To provide the option to complete multiple evaluation methods, relevant to the under-served group, to triangulate findings
7) To ensure that young people from an under-served group have the option to anonymously evaluate PI activities

### Researchers’ positionality

The researchers thought that it was important to reflect on the impact of their positionality within the workshops. The researchers noted that this space was an unfamiliar environment to them. Researchers working within their own cultures are classed as ‘insiders’, whereas those who study cultures different to their own are perceived as ‘outsiders’. Researchers AM and TK did not belong to the same ethic or age group as the young people they were engaging with, and did not live within the same geographical region. AM was concerned that her ‘outsider’ status would influence interactions, however, the young people were extremely welcoming and all academics felt a sense of inclusion when at Eloquent (within the workshops and meetings with the steering group). The researchers particularly valued the steering group as they helped to bridge the perceived ‘insider-outsider’ gap. Within the workshops, the young people listened to each other, and the academics, without judgement and respected individual stories. The researchers felt that all stakeholders (the young people, staff at Eloquent, researchers) had a shared commitment to the Reinvent project. The researchers will take the learnings from Reinvent into future studies in which they work.

### Strengths and limitations

Although this project demonstrates several strengths such as partnering with an under-served group to co-produce the methodological approach taken to evaluating the workshops, there are also limitations that should be identified. The presence of NK and awareness of her dual role among the community members may have biased their evaluation of the project. Furthermore, due to the ways of involvement we could not assure that what was discussed within the workshops would be kept confidential. There is also the potential for the young people to influence each other when completing the ‘Cube’ or when vlogging. As suggested by the young people when evaluating the project, only having four hours to conduct the workshops made some of the activities (e.g. the Cube) feel rushed. Furthermore, there were more females than males involved in the workshops; more efforts may be needed to engage young males. All of the young people were aged 13 years and over, involving younger children may require a different model of working.

## Conclusion

Reinvent has shown that academic institutions and young people from an under-served community can partner to co-design workshops and apply evaluation tools. Creative methods of engagement were key to partnership building with young people, however, these methods need to be carefully co-produced. The use of multiple evaluation methods was beneficial, the young people seemed to particularly value the option to anonymously evaluate the workshops.

### Supplementary Information

**Additional file 1**. GRIPP 2 framework.

## Data Availability

Not applicable.

## Abbreviations

NIHR: National institute for health and care research
PI: Public involvement
REPAG: Race equality public action group
UK: United Kingdom
