# Supplementary material for: An evaluation of a public partnership project between academic institutions and young people with Black African, Asian and Caribbean heritage
Source: Res Involv Engagem. 2024 Mar 19;10:31. doi: 10.1186/s40900-024-00564-4 (PMC10953256; doi:10.1186/s40900-024-00564-4)
Supplement: Supplementary file 1 — Additional file 1. GRIPP 2 framework. [file 40900_2024_564_MOESM1_ESM.docx]

Additional File One: The GRIPP2 Short Form

| Section and topic | Item |
| --- | --- |
| 1: Aim  Report the aim | The aims were:   - To co-design and run two workshops with the young people attending Eloquent (A1) - To co-design an evaluation of each workshop with the young people attending Eloquent (A2) |
| 2: Methods  Provide a clear description of the methods used for patient and public involvement (PPI) in the study | The project developed a steering oversight group that included five young people, one academic (AM), NK and the director of Eloquent. We met in-person once with the objective to meet A1 and A2, however, it quickly became apparent that we would need more time and meetings.  This group met periodically throughout the project with NK also keeping in weekly contact via instant messaging.  At the beginning of the project the steering group met to decide to what extent their involvement would be. The steering group decided that a co-productive approach could be taken as this provided a voice to the young people. The steering group co-produced an agenda for each workshop and the evaluation tools. |
| 3: Results  Outcomes—Report the results of PPI in the study, including both positive and negative outcomes | On the whole, the content of the workshops were relevant to the young people and gave them a platform to share their voice. The co-produced evaluation methods helped researchers to learn from the workshops which may inform how they partner with other under-served groups. |
| 4: Discussion  Outcomes—Comment on the extent to which PPI influenced the study overall. Describe positive and negative effects | The workshops and subsequent evaluations demonstrate the value of partnering with young people from an under-served community. The steering group influenced all aspects of the project and contributed vast amounts of their time to ensure the evaluation tools were relevant and that other young people engaged throughout. |
| 5: Reflections  Critical perspective—Comment critically on the study, reflecting on the things that went well and those that did not, so others can learn from this experience | All young people within the steering group were female and aged 15 and above. Researchers will try to seek a more diverse age range in the future.  Some of the planned activities, which were co-produced with the steering group, changed on the day of the workshops in response to the young peoples’ agendas and preferences; seeking a more diverse steering group may prevent this in the future.  The researchers noted that this space was an unfamiliar environment to them. AM reflected on her positionality within the steering group and Reinvent project in the following way:  It is important to reflect on the impact of my positionality may have on the project and steering group. Researchers working within their own cultures are classed as ‘insiders’, whereas those who study cultures different to their own are perceived as ‘outsiders’. I did not belong to the same ethic or age group as the young people I was engaging with, and did not live within the same geographical region. I am a Caucasian, thirty year old researcher who lives in the Staffordshire Moorlands. I was concerned that as my ‘outsider’ status would influence interactions, however, the young people were extremely welcoming and all academics felt a sense of inclusion when at Eloquent (within the workshops and meetings with the steering group). The young people listened to each other, and the academics, without judgement and respected individual stories. I felt like the staff an Eloquent, and the researchers, has a shared commitment to the Reinvent project which supported the organisation and running of the workshops. I will take the learnings from Reinvent into future studies in which I work. |
